# ALDH1 and ALCAM as emerging biomarkers in personalized rectal cancer therapy

**DOI:** 10.1590/0102-67202025000051e1920

**Published:** 2026-02-16

**Authors:** Jurandir Marcondes RIBAS-FILHO, Efstathia N DOELKEN, Sudipta TRIPATHI, Bülent POLAT, Reinhard LISSNER, Thomas BÖELDICKE, Carmen Austrália Paredes Marcondes RIBAS, Osvaldo MALAFAIA, Martin GASSER, Ana Maria WAAGA-GASSER

**Affiliations:** 1 Faculdade Evangélica Mackenzie do Paraná – Curitiba (PR), Brazil.; 2 University of Wuerzburg, Molecular Oncology and Immunology, Department of Surgery I – Wuerzburg, Bavaria, Germany.; 3 University of Massachusetts Chan Medical School, Department of Medicine, Division of Renal Medicine – Worcester (MA) USA.; 4 University of Wuerzburg, University Hospital, Department of Radiation Oncology – Wuerzburg, Bavaria, Germany.; 5 Helmholtz Centre for Infection Research – Braunschweig, Germany.; 6 Harvard Medical School, Brigham and Women’s Hospital, Department of Medicine, Renal Division – Boston (MA), USA.

**Keywords:** Rectal cancer, Biomarkers, Aldehyde dehydrogenase, Activated leukocyte cell adhesion molecule, Neoplastic stem cell, Chemoradiotherapy, Câncer retal, Biomarcadores, Aldeído desidrogenase, Molécula de adesão de leucócito ativado, Células-tronco neoplásicas, Quimioradioterapia

## Abstract

**Background:**

The preoperative evaluation of serum tumor markers provides valuable prognostic and therapeutic insights in solid malignancies. Although not diagnostic by themselves, increased preoperative concentrations often reflect greater tumor burden, advanced disease stage, and unfavorable clinical outcomes.

**Aims:**

The aim of this study was to investigate the expression and diagnostic-prognostic potential of the tumor markers aldehyde dehydrogenase 1 (ALDH1) and activated leukocyte cell adhesion molecule (ALCAM) in the blood of rectal cancer patients under the influence of short- or long-term radio-/chemoradiotherapy (RTx/RCTx).

**Methods:**

Serum samples taken from patients with rectal carcinoma (n=164) at different time points during and after RTx/RCTx were retrospectively examined to determine whether these markers could predict disease progression and long-term survival.

**Results:**

Kaplan-Meier analysis confirmed the prognostic relevance of the Union for International Cancer Control (UICC) staging, while no significant associations were observed between serum levels of the investigated biomarkers and individual patient or tumor characteristics such as age, sex, or tumor stage. Overall, ALCAM and ALDH1 in this limited patient cohort exhibited elevated serum levels compared with healthy controls, and tumor tissues demonstrated stage-dependent increases in marker expression (UICC III/IV versus I/II).

**Conclusions:**

Serum concentrations of ALCAM and ALDH1 were significantly elevated in our patient cohort with rectal cancer but showed no significant correlation with tumor stage or survival, whenever serum samples were obtained either during or after neoadjuvant and adjuvant therapy, which may be particularly due to the limited number of studied subjects. Although their prognostic utility remains limited, their consistent elevation in cancer patients underscores their potential value in early detection or as components of a broader biomarker panel.

## ARTICLE HIGHLIGHTS

Rectal cancer continues to represent a major public health issue worldwide, with incidence rising progressively with advancing age.

The preoperative evaluation of serum tumor markers offers significant prognostic and therapeutic insights in patients with solid neoplasms.

Carcinoembryonic Antigen (CEA) remains a well-established prognostic indicator in colorectal cancer.

Cancer stem cells (CSCs) have emerged as pivotal elements in the onset, progression, and recurrence of malignant tumors.

Multiple markers have been proposed in CRC as indicative of CSC activity, including CD44, CD24, CD133, CD166, ALCAM, EpCAM, LGR5, and ALDH1.

## Summary

**CENTRAL MESSAGE:**

Rectal cancer is a major global health concern, with incidence increasing alongside aging populations. Although the overall 5-year survival rate is only slightly higher than that of colon cancer, stage-specific outcomes remain comparable between the two entities. Nevertheless, patients with rectal carcinoma exhibit a greater propensity for locoregional recurrence and, in many cases, distant metastatic spread, despite continuous advances in multimodal treatment strategies.

**PERSPECTIVES:**

The evaluation of serum biomarkers such as aldehyde dehydrogenase 1 (ALDH1) and activated leukocyte cell adhesion molecule (ALCAM) offers new insights into the biological behavior of rectal cancer. Their analysis may contribute to refining prognostic assessment and therapeutic monitoring, ultimately advancing the development of more individualized approaches for patients undergoing neoadjuvant and adjuvant therapies.

## VISUAL ABSTRACT

**Figure:**
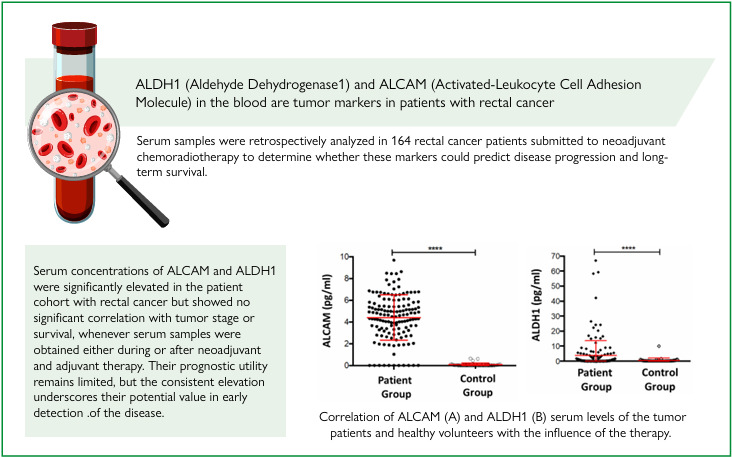

## RESUMO

**Racional:**

A avaliação pré-operatória de marcadores tumorais séricos fornece valiosos insights prognósticos e terapêuticos em neoplasias sólidas. Embora não sejam diagnósticos por si só, o aumento das concentrações pré-operatórias geralmente reflete maior carga tumoral, estágio avançado da doença e desfechos clínicos desfavoráveis.

**Objetivos:**

Investigar a expressão e o potencial diagnóstico-prognóstico dos marcadores tumorais ALDH1 e ALCAM no sangue de pacientes com câncer de reto sob influência de radio/radioquimioterapia de curta ou longa duração (RTx/RCTx).

**Métodos:**

Amostras de soro retiradas de pacientes com carcinoma retal (n=164) em diferentes momentos durante e após RTx/RCTx foram examinadas retrospectivamente para determinar se esses marcadores poderiam prever a progressão da doença e a sobrevida em longo prazo.

**Resultados:**

A análise de Kaplan-Meier confirmou a relevância prognóstica do estadiamento da UICC, enquanto não foram observadas associações significativas entre os níveis séricos dos biomarcadores investigados e as características individuais do paciente ou do tumor, como idade, sexo ou estágio tumoral. No geral, ALCAM e ALDH1 nesta coorte limitada de pacientes exibiram níveis séricos elevados em comparação com controles saudáveis, e os tecidos tumorais demonstraram aumentos dependentes do estágio na expressão do marcador (UICC III/IV versus I/II).

**Conclusões:**

As concentrações séricas de ALCAM e ALDH1 foram significativamente elevadas nesta coorte de pacientes com câncer retal, mas não mostraram correlação significativa com o estágio ou sobrevida do tumor, sempre que amostras de soro foram obtidas durante ou após a terapia neoadjuvante e adjuvante, o que pode ser particularmente devido ao número limitado de indivíduos estudados. Embora sua utilidade prognóstica permaneça limitada, sua elevação consistente em pacientes com câncer ressalta seu valor potencial na detecção precoce ou como componentes de um painel de biomarcadores mais amplo.

## INTRODUCTION

 Rectal cancer continues to represent a major public health issue worldwide, with incidence rising progressively with advancing age. According to current Surveillance, Epidemiology, and End Results (SEER) statistics from the American Cancer Society, the 5-year survival rate for rectal carcinoma (67%) remains only marginally higher than that observed for colon cancer (63%). Despite this small difference, the stage-specific survival outcomes between both entities are essentially equivalent^1^. Nonetheless, patients with rectal cancer present a greater tendency toward locoregional recurrence and, in several studies, a higher frequency of distant metastatic spread, even in the era of multimodal treatment strategies^14,19^. 

 Although serum tumor markers lack absolute specificity and sensitivity, they continue to play a valuable role in the management of patients with solid tumors. These markers assist in refining prognostic assessment, guiding adjuvant therapeutic decisions, and monitoring patients after surgical intervention^17,21,25^. 

 The preoperative evaluation of serum tumor markers offers significant prognostic and therapeutic insights in patients with solid neoplasms. Although these biomarkers are not diagnostic per se, elevated concentrations prior to surgery frequently reflect a greater tumor load, more advanced stages, and an unfavorable clinical course. Individuals presenting with increased preoperative marker levels tend to experience poorer outcomes, characterized by reduced disease-free survival (DFS) and overall survival. Such parameters serve as valuable baselines for postoperative follow-up, since persistently high values after resection may indicate residual disease or the presence of undetectable micrometastases, thereby supporting decisions regarding adjuvant treatment^24,27^. 

 Moreover, serial measurements can aid in evaluating therapeutic efficacy and in the early detection of recurrence. Carcinoembryonic antigen (CEA) remains a well-established prognostic indicator in colorectal cancer (CRC) and is routinely measured before surgery in affected patients^3,8^. In gastrointestinal tumors, the combination of CEA and carbohydrate antigen 19-9 (CA19-9) enhances risk stratification^5,6^, whereas cancer antigen-125 (CA-125) serves as a marker of stage and resectability in ovarian malignancies. Despite inherent limitations in sensitivity and specificity, preoperative serum markers continue to play an important role in refining prognosis, guiding adjuvant therapy, and facilitating long-term surveillance. In the current era of multimodal and individualized oncologic treatment, there is a growing demand for comprehensive soluble marker profiles beyond the conventional CEA and CA19-9 determinations^3,8^. 

 Cancer stem cells (CSCs) have emerged as pivotal elements in the onset, progression, and recurrence of malignant tumors^8^. A distinctive feature of this subpopulation is its pronounced resistance to chemotherapeutic agents. This resistance is partly attributed to the overexpression of drug efflux transporters, which actively remove cytotoxic compounds — such as alkylating agents — from the intracellular environment, thereby reducing the therapeutic efficacy of chemotherapy. Over recent years, considerable research has focused on identifying cell surface markers and molecular signaling pathways that differentiate CSCs from both normal somatic and physiological stem cells. Such discoveries aim to enable the selective detection and targeted eradication of tumor-initiating cells. Multiple markers have been proposed in CRC as indicative of CSC activity^12^, including cluster of differentiation 44 (CD44), CD24, CD133, CD166, activated leukocyte cell adhesion molecule (ALCAM), epithelial cell adhesion molecule (EpCAM), leucine-rich repeat-containing G-protein-coupled receptor 5 (LGR5), and aldehyde dehydrogenase 1 (ALDH1)^15,16,20^. These molecules have positioned CSCs as promising targets for innovative approaches in cancer screening, early diagnosis, prevention, and treatment, particularly in CRC^22,23^. 

 ALDH1 functions as a key enzyme in the metabolism of intracellular aldehydes and is widely used as a marker for CSCs, which are responsible for the oxidation of a wide range of toxic endogenous and exogenous aldehydes. High expression levels of ALDH1 are associated with a poor prognosis for many different solid tumors, including esophageal, breast, lung, stomach, and ovarian cancers^1^. However, increased expression of ALDH1 was also observed in colorectal tumors, although, paradoxically, it showed a downward trend in some studies (which focused on advanced stages of the disease)^10^. In numerous studies, ALDH1 is an important independent prognostic marker in CRC^7^. In another study, the expression of ALDH1 was investigated in connection with various clinical and pathological parameters. A strong association between protein expression and tumor stage (T), lymph node status (N), and tumor differentiation (G) was observed. The patient’s age appeared to be irrelevant to prognosis^4^. Furthermore, ALDH1-positive cells in CRC tumors exhibit enhanced tumorigenicity, resistance to chemotherapy, and the capacity to initiate metastases, correlating with advanced disease stage, increased recurrence rates, and poorer survival outcomes. This indicates its utility as a prognostic biomarker and a target for CSC-directed therapies. 

 ALCAM (CD166) is a cell adhesion molecule that plays roles in cell-cell interactions and tumor progression and plays a role in maintaining hematopoietic stem cell populations in the bone marrow^11^. Altered ALCAM expression has been noted in multiple cancers, including melanoma, prostate, esophageal, breast, and urothelial carcinomas^18^. While some tumors show ALCAM upregulation, others exhibit downregulation. In CRC, membranous ALCAM expression correlates with shorter survival and strong cytoplasmic expression, and its expression has been linked to enhanced invasive and metastatic capabilities^26^. Upregulation of ALCAM appears to be an early event in the development of colonic tumors^26^. Although results vary, increased ALCAM expression generally associates with more aggressive tumor phenotypes and worse clinical prognosis. Emerging data also suggest that ALCAM contributes to maintaining the CSC niche, further implicating it in tumor progression and treatment resistance. 

 ALDH1 and ALCAM serve as important markers of tumor stem cell activity in CRC, providing prognostic information related to tumor aggressiveness, metastatic potential, and therapeutic resistance. Their expression levels may guide clinical decision-making, and they represent potential targets for novel therapeutic interventions aimed at improving patient outcomes. Further research is warranted to validate their prognostic utility and to develop effective CSC-targeted therapies in rectal cancer. 

 This study focused on the expression and diagnostic/prognostic potential of the tumor markers ALDH1 and ALCAM, in rectal cancer, individually or in combination. For this purpose, serum levels were determined to retrospectively investigate whether predictions about the course of the disease and long-term patient survival could have been derived from these. 

## METHODS

 This retrospective study evaluated a cohort of 164 patients (42 women and 122 men) with histologically confirmed rectal carcinoma who received treatment at the University Hospital of Wuerzburg, Germany. Neoadjuvant management consisted of either short-course radiotherapy (RTx) or long-course chemoradiotherapy (RCTx). The short-course regimen comprised five daily fractions of 5 Gy (total dose 25 Gy) directed at the primary rectal lesion and surrounding pelvic tissues over 1 week. The long-course protocol delivered 28 fractions of 1.8 Gy (total dose 50.4 Gy) over 6 weeks, combined with systemic chemotherapy — either two cycles of 5-fluorouracil (5-FU, 100 mg/m^2^/day on days 1–5 during weeks 1 and 5) or a combined regimen of 5-FU (250 mg/m^2^, days 1–14 and 22–35) with oxaliplatin (50 mg/m^2^ on days 1, 8, 22, and 29). All primary tumor resections were performed exclusively at the Department of Surgery of the same institution. After hospital discharge, postoperative surveillance was conducted in the Surgical Polyclinic, affiliated oncology units, or outpatient departments of the Comprehensive Cancer Center. Follow-up included physical examination, blood collection, chest radiography, abdominal ultrasonography, and cross-sectional imaging (computed tomography or magnetic resonance imaging), according to German tumor center guidelines. Clinical and pathological information was extracted from institutional medical records using the International Classification of Diseases (ICD) coding system. Key variables recorded were sex, age, and tumor stage, classified according to the Union for International Cancer Control (UICC) criteria. Serum and tissue samples were obtained with informed patient consent from the Tumor Tissue and Sera Bank of the Interdisciplinary Bank of Biomaterials and Data Wuerzburg (IBDW)^9^. Blood samples were collected at multiple time points — before and during RCTx, prior to surgery, and during postoperative follow-up — by the Department of Radio-Oncology, the Surgical Clinic, and associated oncology centers^9^. 

 Protein concentrations of ALCAM and ALDH1 were determined in serum samples using commercially available enzyme-linked immunosorbent assay (ELISA) kits, following the manufacturers’ protocols. For ALCAM, the Cloud Clone Corp Human ALCAM ELISA Kit (Catalog #SEA002; sample volume: 2×100 μL) was employed, while ALDH1 levels were measured using the Cloud Clone Corp Human ALDH1 ELISA Kit (Catalog #KA1696; sample volume: 2×100 μL). Sera from 39 healthy individuals served as controls for baseline comparison. All assays were performed in duplicate according to standard procedures, and absorbance values were recorded at 450 nm using a Dynatech Laboratories ELISA reader (Sullyfield, USA). Final concentrations were expressed in ng/ml and pg/ml, respectively. 

 Immunofluorescent analysis was performed on cryostat sections obtained from snap-frozen rectal tumor specimens. Primary antibodies specific for ALCAM (clone EPR4421, dilution 1:300, Abcam, catalog #ab109489) and ALDH1 (clone EPR1933Y, dilution 1:100, Abcam, catalog #ab215996) were used. Secondary antibodies conjugated with Cy3 (indocarbocyanine) and AlexaFluor 488 fluorochromes were sourced from Jackson ImmunoResearch (West Grove, PA, USA). Tissue sections were fixed in acetone, permeabilized with methanol, and subsequently incubated overnight at 4°C in a humidified chamber with primary antibodies diluted in TBS containing 0.5% bovine serum albumin (BSA). After washing, samples were incubated for 30 min at room temperature with the appropriate fluorochrome-labeled secondary antibody under identical humidified conditions. The stained slides were visualized using an Olympus BX51 fluorescence microscope equipped with CellSens Dimension software. Quantitative evaluation was performed independently by two blinded investigators, who calculated the mean number of positively stained cells in three consecutive high-power fields. In cases of disagreement, the final score was reached by consensus discussion. The study was approved by the Ethics Committee of the Institution. 

### Statistical analysis

 All statistical analyses and figure generation were performed using GraphPad Prism version 6 and SPSS software version 12.0 (University of Wuerzburg, Germany). A p-value below 0.05 was considered statistically significant (*), while values <0.01 were interpreted as highly significant (**). Comparative analyses were conducted using a combination of statistical tests, including the ꭓ^2^ test for categorical variables, unpaired t-test, and one-way analysis of variance (ANOVA) for continuous variables. Correlations between the expression of ALCAM and ALDH1 were assessed through univariate analysis. Survival outcomes were examined by the KaplanMeier method, and differences between groups were evaluated with the log-rank test. Tumor-related survival (TRS) was defined as the interval between diagnosis and either tumorrelated death or last follow-up, while recurrence-free survival (RFS) corresponded to the time from diagnosis to the first documented recurrence. Data from patients who were alive or recurrence-free at the last follow-up were treated as censored observations in the survival analyses. 

## RESULTS

**Figure 1:**
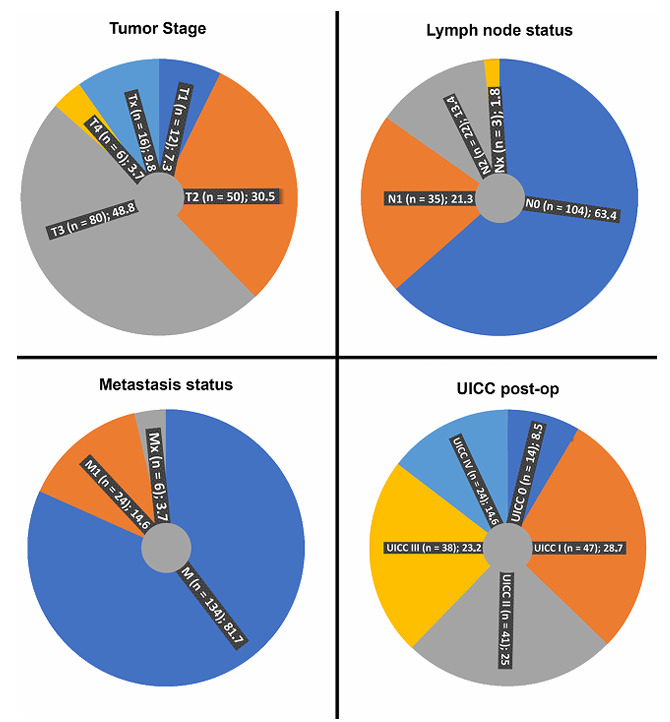
Patient stratification by tumor stage, nodal status, metastasis, and postoperative the Union for International Cancer Control stage.

 A total of 164 patients diagnosed with rectal carcinoma were included in the present analysis. The cohort (Figure 1) exhibited a marked predominance of males, representing 74.4% (122/164) of the population, compared with 25.6% (42/164) females — a ratio of approximately three to one. Because of this gender imbalance, and the limited number of female participants, all cases were analyzed collectively without sex-based stratification. Patient ages ranged from 31.4 to 83.6 years, with a median of 64.3 years. The median follow-up period was 45 months, during which 53 patients (32.3%) succumbed to their disease. Approximately onethird (37.2%) of the subjects presented with early-stage tumors (UICC 0–I) characterized by Tis/T1/T2, N0, and M0 at diagnosis. Conversely, 24 individuals (14.6%) were found to have distant metastases (UICC IV), indicating a predominance of locally advanced disease at presentation. 

**Figure 2:**
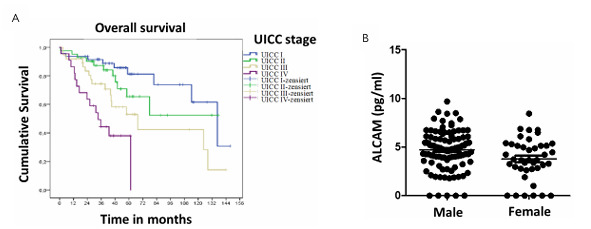
(A) Influence of the Union for International Cancer Control stage on overall survival in patients with rectal cancer (Kaplan-Meier curve); (B) correlation of the Activated Leukocyte Cell Adhesion serum levels with the gender groups.

**Figure 3:**
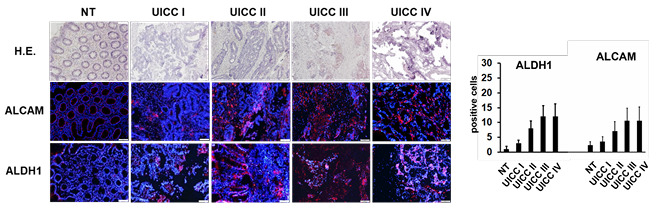
Representative examples of the analyzed primary rectal cancers from study patients for Activated Leukocyte Cell Adhesion and Aldehyde Dehydrogenase 1 expression using staining: on top row, hematoxylin & eosin (H.E.) of corresponding tissue sections; below staining (100x): nuclei (DAPI, blue), positive marker expression (AlexaFluor 488, red). NT: no tumor; UICC: Union for International Cancer Control; ALDH1: Aldehyde Dehydrogenase 1; ALCAM: Activated Leukocyte Cell Adhesion Molecule.

**Figure 4:**
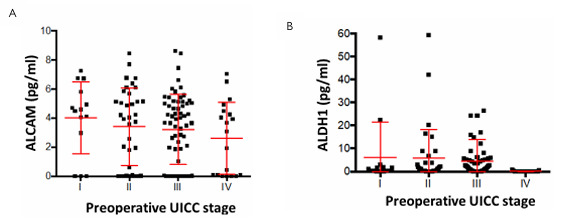
Comparison of Activated Leukocyte Cell Adhesion Molecule (A) and Aldehyde Dehydrogenase 1 (B) serum levels with Union for International Cancer Control stages of the patients. ALDH1: Aldehyde Dehydrogenase 1; ALCAM: Activated Leukocyte Cell Adhesion Molecule; UICC: Union for International Cancer Control.

 The prognostic relevance of key clinical and pathological parameters in patients with rectal cancer was assessed using the Kaplan-Meier survival analysis. No statistically significant differences in TRS were detected when stratified by gender or age. Conversely, UICC staging demonstrated a strong and statistically significant association with overall survival (p<0.001, Figure 2A), reaffirming the critical prognostic value of this classification system and confirming the representativeness of the studied cohort. Immunofluorescence evaluation of the primary tumor specimens revealed progressively increased marker expression in advanced disease stages (UICC III–IV) compared with early stages (UICC I–II) and with minimal expression in normal rectal mucosa (Figures 3 and 4). These findings emphasize the stage-dependent biological behavior of the investigated biomarkers. 

 For ALCAM, males showed significantly higher expression than females (p=0.013; Figure 2B). For ALDH1, no significant correlation was found (data not shown). 

**Figure 5:**
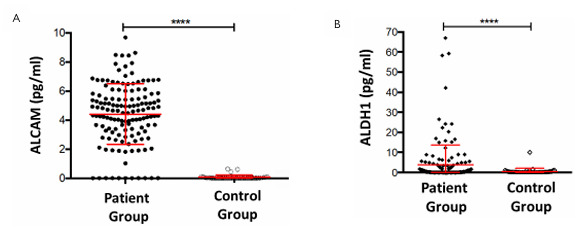
Correlation of Activated Leukocyte Cell Adhesion Molecule (A) and Aldehyde Dehydrogenase 1 (B) serum levels of the tumor patients and healthy volunteers under the influence of the therapy. ALDH1: Aldehyde Dehydrogenase 1; ALCAM: Activated Leukocyte Cell Adhesion Molecule.

 Correlation between ALCAM and ALDH1 with overall patient survival and DFS was obtained using the Kaplan-Meier method. There was no influence of serum expression levels of ALCAM and ALDH1 on both overall survival and DFS of the patients. The correlation of serum levels for both markers in patients with rectal cancer under the influence of RTx/RCTx and in healthy volunteers showed significantly higher serum levels (p<0.0001, two-sided unpaired t-test, mean: 4.4 in tumor patients versus 0.06 pg/ml in controls and 3.9 versus 0.6 pg/ml, respectively) (Figure 5). 

**Figure 6:**
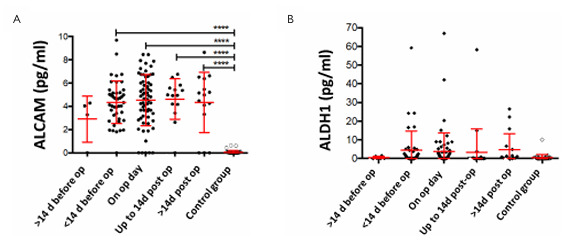
Correlation of Activated Leukocyte Cell Adhesion Molecule (A) and Aldehyde Dehydrogenase 1 (B) serum levels at different time intervals before and after removal of the tumor (healthy volunteers for comparison). The patients were divided into five groups: (i) blood sampling more than 2 weeks before surgery, (ii) within 2 weeks before surgery, (iii) on the day of surgery (before the start of the removal of the tumor), (iv) within 2 weeks after surgery, and (v) more than 2 weeks after surgery and compared with healthy volunteers. The patients were divided into two groups: (i) those who had received short-term RTx (1 to ≤5 weeks of neoadjuvant therapy) and those who had undergone long-term RCTx (≥5 weeks). op: operation; ALDH1: Aldehyde Dehydrogenase 1; ALCAM: Activated Leukocyte Cell Adhesion Molecule.

 There was no significant decrease in elevated ALCAM values after surgery (Figure 6). Moderately elevated ALDH1 values could be detected preferentially before and also after surgical removal of the tumors (less than 2 weeks before and after surgery), but also after surgical intervention (more than 14 days after surgery). 

**Figure 7:**
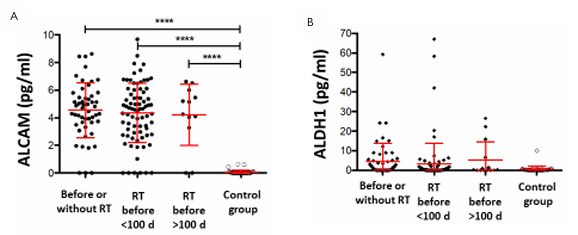
Correlation of Activated Leukocyte Cell Adhesion Molecule (A) and Aldehyde Dehydrogenase 1 (B) serum levels of the tumor patients and healthy volunteers under the influence of the therapy. ALDH1: Aldehyde Dehydrogenase 1; ALCAM: Activated Leukocyte Cell Adhesion Molecule; RTx: radiotherapy.

 Elevated ALCAM levels in the tumor patients as compared to controls were not influenced by neoadjuvant therapy (Figure 7A). The same observation was also made when examining the influence of neoadjuvant therapy on elevated serum ALDH1 levels (Figure 7B). 

## DISCUSSION

 In the present investigation, serum concentrations of the candidate biomarkers ALCAM and ALDH1 were evaluated in a cohort of 164 patients newly diagnosed with rectal carcinoma. The objective of the study was to determine potential correlations between these markers and the clinicopathological parameters, including overall survival, DFS, and the therapeutic response to neoadjuvant RCTx. 

 In accordance with previously reported epidemiological data, the present cohort exhibited a predominance of male individuals, with a median age of 64.3 years, mirroring demographic characteristics commonly described in rectal cancer populations. Disease staging at diagnosis encompassed both early and advanced presentations, with roughly 15% of cases demonstrating distant metastatic involvement. The KaplanMeier survival analysis reaffirmed the prognostic relevance of the UICC classification, emphasizing its robustness as a principal framework for outcome prediction in rectal carcinoma. Notably, neither of the two investigated serum biomarkers showed a significant association with age, tumor stage, or sex, with the exception of ALCAM, which presented significantly higher expression levels in male patients (p=0.013, p<0.05). The absence of correlation between serum biomarker levels and tumor stage indicates that circulating concentrations may not accurately mirror the actual tumor burden, differing from their tissue expression patterns, which were markedly increased in advanced lesions. Such discrepancies between serum and tissue findings are consistent with prior reports suggesting that systemic biomarker levels can be modulated by factors including protein clearance, receptor shedding, or therapy-induced alterations^4,6^. 

 The survival analysis did not demonstrate any significant relationship between ALCAM or ALDH1 expression and overall or DFS. Although elevated serum levels of these markers have been linked to reduced survival and increased recurrence in several other malignancies^1,2,5,10,13^, such associations were not observed in this cohort. These findings suggest that, when assessed individually, serum ALCAM and ALDH1 may possess limited prognostic utility in rectal cancer. However, their combined evaluation with other molecular markers or within defined patient subgroups could still provide clinically relevant insights. Immunohistochemical studies have demonstrated that elevated ALDH1 expression within tumor tissues is associated with higher T and N stages of rectal carcinoma^4^, as well as with the UICC classification and concurrent expression of ALCAM^4,26^. These relationships were corroborated by the findings from our immunofluorescence analyses, which revealed comparable patterns of marker upregulation. 

 Serum concentrations of ALCAM and ALDH1 were found to be elevated, aligning with the overexpression patterns previously reported in various tumor types^10,18,26^. This result suggests a tumor-driven dysregulation of these biomarkers, seemingly unaffected by surgical resection or neoadjuvant treatment, as their levels remained relatively stable both before and after surgery. 

 Taken together, the present results indicate that serum expression of ALCAM and ALDH1 may represent potential biomarkers indicative of rectal cancer. Nevertheless, additional investigations involving larger, treatment-stratified cohorts are necessary to confirm these observations and to elucidate both the prognostic and predictive significance of these markers, as well as the biological mechanisms underlying their modulation in circulation. Furthermore, longitudinal evaluation of ALCAM and ALDH1 could yield valuable information regarding tumor dynamics, therapeutic response, and tissue repair mechanisms throughout the course of rectal cancer management. 

 This study presents certain limitations, including its retrospective design, the imbalance in gender distribution, and the relatively small number of control subjects. In addition, the heterogeneity of the cohort — particularly regarding the inclusion of patients who received or did not receive neoadjuvant RCTx, as well as the variability in the timing of blood collection — may have influenced serum marker levels. These factors should be carefully controlled in future prospective investigations. Although accumulating evidence supports the prognostic potential of ALCAM, several challenges remain before its incorporation into routine clinical practice. Standardization of analytical techniques, establishment of reliable cutoff values, and validation in larger prospective cohorts with extended longitudinal follow-up (exceeding 200 days across neoadjuvant, adjuvant, and surgical phases) are essential. Furthermore, the interrelationship between these biomarkers and their combined prognostic significance warrants continued and detailed exploration. 

## CONCLUSIONS

 Serum concentrations of ALCAM and ALDH1 were found to be elevated in patients with rectal cancer compared to healthy individuals. Notably, these alterations showed no significant association with tumor stage or survival outcomes, differing from the expression patterns observed in tumor tissues both in the present analysis and in previously published studies. In addition, the observed relationship between ALCAM and other biomarkers points to interconnected molecular pathways that merit further investigation. Collectively, these findings highlight the complexity and multifactorial nature of interpreting serum biomarkers in rectal cancer. Their levels remained stable across treatment phases, including after surgery and RTx, suggesting that these markers are not acutely modulated by therapy and may reflect persistent tumor-associated or microenvironmental signaling. While their prognostic utility is limited, their consistent elevation in cancer patients highlights their potential role in early detection or as part of a broader biomarker panel. Further studies are warranted to evaluate their role in cancer stemness and treatment resistance in longitudinal, treatment-stratified cohorts. 

## Data Availability

The datasets generated and/or analyzed during the current study are available from the corresponding author upon reasonable request.
